# Current management strategies for the pain of elderly patients with burning mouth syndrome: a critical review

**DOI:** 10.1186/s13030-019-0142-7

**Published:** 2019-01-31

**Authors:** Trang T. H. Tu, Miho Takenoshita, Hirofumi Matsuoka, Takeshi Watanabe, Takayuki Suga, Yuma Aota, Yoshihiro Abiko, Akira Toyofuku

**Affiliations:** 1 Department of Psychosomatic Dentistry, Graduate School of Medical and Dental sciences, Tokyo Medical and Dental University, 1-5-45 Yushima, Bunkyo, Tokyo, 113-8549 Japan; 2 Division of Disease Control and Molecular Epidemiology, Department of Oral Growth and Development, School of Dentistry, Health Sciences University of Hokkaido, Hokkaido, Japan; 3 Division of Oral Medicine and Pathology, Department of Human Biology and Pathophysiology, School of Dentistry, Health Sciences University of Hokkaido, Hokkaido, Japan

**Keywords:** Burning mouth syndrome, Medically unexplained oral symptoms, Management strategies, Elderly, Neuromodulators, Oral facial pain, Psychotherapy

## Abstract

Burning Mouth Syndrome (BMS), a chronic intraoral burning sensation or dysesthesia without clinically evident causes, is one of the most common medically unexplained oral symptoms/syndromes. Even though the clinical features of BMS have been astonishingly common and consistent throughout the world for hundreds of years, BMS remains an enigma and has evolved to more intractable condition. In fact, there is a large and growing number of elderly BMS patients for whom the disease is accompanied by systemic diseases, in addition to aging physical change, which makes the diagnosis and treatment of BMS more difficult. Because the biggest barrier preventing us from finding the core pathophysiology and best therapy for BMS seems to be its heterogeneity, this syndrome remains challenging for clinicians. In this review, we discuss currently hopeful management strategies, including central neuromodulators (Tricyclic Antidepressants - TCAs, Serotonin, and Norepinephrine Reuptake Inhibitors - SNRIs, Selective Serotonin Reuptake Inhibitors - SSRIs, Clonazepam) and solutions for applying non-pharmacology approaches. Moreover, we also emphasize the important role of patient education and anxiety management to improve the patients’ quality of life. A combination of optimized medication with a short-term supportive psychotherapeutic approach might be a useful solution.

## Introduction

**Fig. 1:**
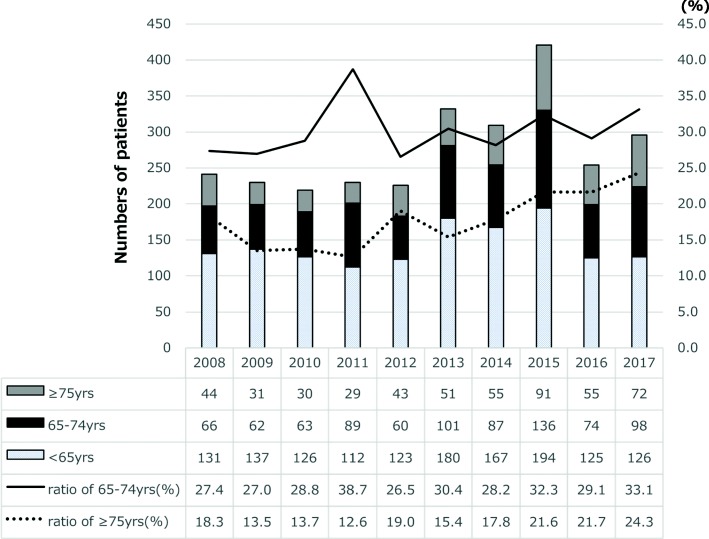
The proportion of elderly among first-visit Burning Mouth Syndrome patients over the past 10 years (2008–2017)

Burning Mouth Syndrome (BMS), also called “stomatodynia” or “glossodynia”, is one of the most common medically unexplained oral symptoms/syndromes (MUOS) [1, 2]. Over centuries, a large number of BMS studies have been conducted about the pathophysiology [3–5], but so far with limited knowledge because of its heterogeneity [6, 7]. Although the clinical features of BMS have been astonishingly common and consistent throughout the world for hundreds of years, an ultimate treatment strategy has not been established [8–11]. At the Department of Psychosomatic Dentistry, Dental Hospital, Tokyo Medical and Dental University (TMDU), Japan, we have around 250 new BMS patients every year and currently treat 4–5000 outpatients. Among them, approximately 55% are over 65 years old. Because the majority of elderly patients are accompanied by systemic diseases and contraindications of tricyclic antidepressant, the first line in management of BMS, the population aging poses challenges in patient management [12]. (Fig. 1) The actual situation makes the diagnosis and treatments of BMS more complex and difficult. A recent study in the United Kingdom shows the profound financial impact of persistent orofacial pain on patients’ lives, in which the ‘hidden economical-social cost’ was calculated around 3000 GBP (Great Britain Pound) per year [13]. It has attracted attention and controversy as to HOW we should manage this syndrome in the elderly[14]. In this review, we discuss real-world, useful strategies for the management of patients with BMS, especially the elderly.

## Overview of burning mouth syndrome

### Definition

The International Association for the Study of Pain (IASP) presents BMS as “a chronic condition characterized by a burning sensation of the oral mucosa for which no cause can be found” [1]. The International headache society (IHS) defines BMS as “an intraoral burning sensation or dysesthesia, recurring daily for more than 2 hours per day over more than 3 months, without clinically evident causative lesions” [15]. This definition concretely shows the duration of daily and consecutive symptoms, thus it was preferred for use in diagnosis. The common finding among the definitions of BMS is the involvement of no obvious clinical causative lesions. However, the term BMS is sometimes used to describe an oral burning sensations that is induced by several local or systemic conditions, also called “secondary” BMS, instead of an “exact” oral pain/dysesthesia condition with unknown origin or “primary” BMS. This inconsistency indicates that although the diagnostic criteria for BMS have become more sophisticated they remain a little rough. They can include many causative factors and heterogeneous patients, because of the lack of accurate biomarkers and little knowledge of pathophysiology [7, 16].

### Epidemiology

There are several epidemiology studies that include “secondary” BMS while only a few are conducted on “exact” BMS. Overall, the prevalence of BMS in the adult population has been reported to be between 0.7 and 3.7% [17, 18]. The syndrome usually occurs in middle-aged and elderly patients more often than in children and adolescents, and female predominance has been reported (female: male = 7:1) [19]. The relevance of psychiatric disorders in BMS is remains to be clarified, but one study reported that about 50% of BMS patients have specific psychiatric diagnoses, 60% of whom are diagnosed with mood disorders [20]. Overlap with other MUOS (atypical odontalgia, phantom bite syndrome, oral cenesthopathy) should be also carefully considered. BMS is sometimes comorbid with atypical odontalgia in the same patient, which contributes to a more intensively painful experience [21].

### Pathophysiology

BMS is a syndrome of unknown causes for which the etiology and pathological origin are under debate [7, 16]. Patients often have been regarded as having psychogenic conditions [22]. Although many attempts have been made to clarify the relation between BMS and psychological factors, the relation remains unclear [20, 23, 24].

The majority of BMS patients are postmenopausal women, thus the association with female hormones has been proposed [25]. Furthermore, a study reported that because BMS patients frequently suffer from taste disturbance and other similar problems, dysfunction of the chorda tympani nerve may be involved [26]. Other researchers support the hypothesis that BMS may be a neuropathic pain involving the central nervous system [4, 9, 27]. It is probably true that some central sensitization might be related to BMS, as are other functional somatic syndromes [28, 29], however, recent evidence shows its limitations, especially for elderly patients [30, 31]. In addition, the pathophysiology should be considered not only as a pure painful sensation but also as oral discomfort that includes dysgeusia and subjective dry mouth [32, 33], which seems to be more common in the elderly.

**Fig. 2:**
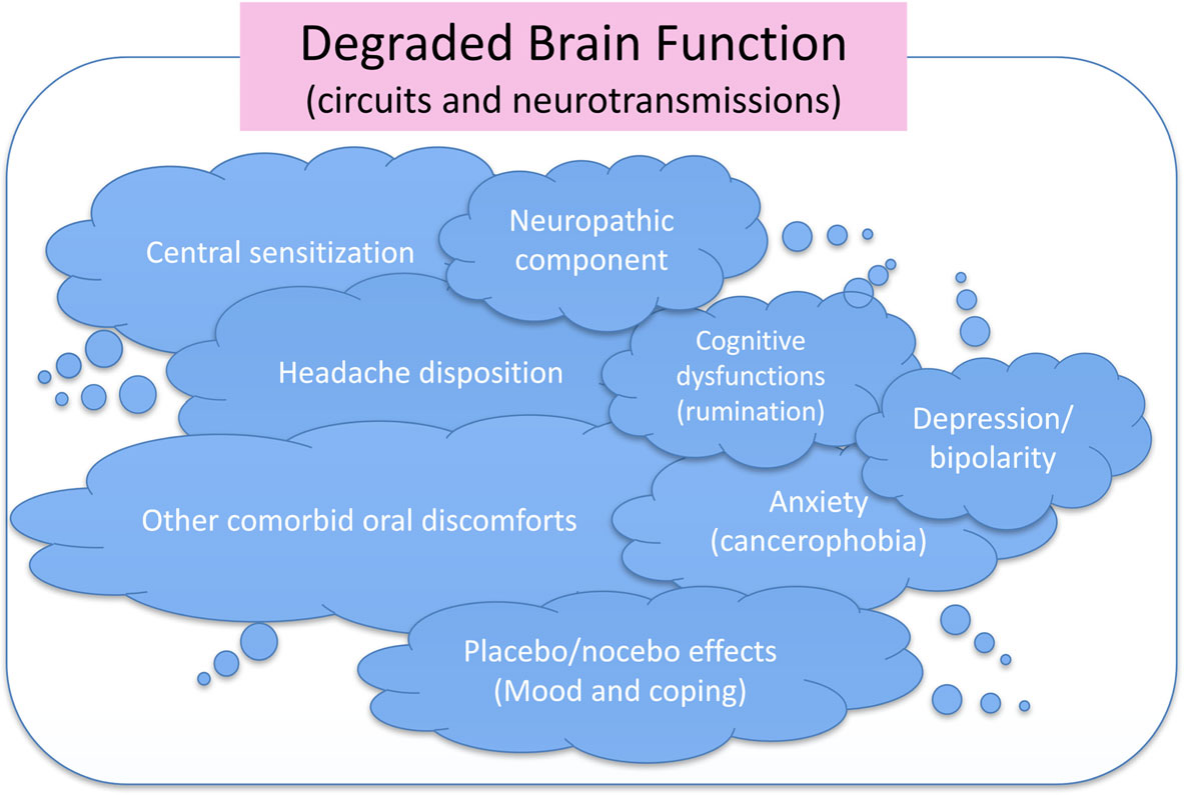
Causative components of Burning Mouth Syndrome

In this review, we assume that the etiology and pathophysiology of BMS might not be that simple, but rather a complex, multifactorial condition. BMS symptoms seem to represent something of an amalgam of various factors in the same patient. (Fig. 2) From a clinical viewpoint, the efficacy of some antidepressants [2, 3, 5, 9–11] might be the best evidence showing the relation to dysregulation of some neurotransmitters, including dopamine nervous systems [7], which probably affect complex neurological networks [29]. In future study, neuroimaging will play a promising, key-role in clarifying the mechanisms of the central nervous system [34–37].

## Diagnosis based on clinical features

The diagnosis of BMS remains challenging because it shares symptoms with several conditions, such as Candida infection, allergy, or nutritional deficiency. In real-life clinical situations, instead of using the classification-based criteria of ICHD or IASP clinicians usually do differential diagnosis to rule out other possible related conditions [3, 10, 11]. To address how to do more precise BMS diagnosis, we suggest in this review that some classical clinical features be added to the official criteria proposed by ICHD and IASP. (Table 1) These clinical features of BMS might be helpful for decreasing the time to diagnosis and improving accuracy.

**Table 1:** Characteristic symptoms of patients with Burning Mouth Syndrome

1	Pain may immigrate or spread independent of the anatomy of peripheral nerves (facial skin is not usually affected)
2	Spontaneous pain that worsens as the day progresses
3	No pain during eating, sleeping or concentrating on something
4	Symptom relief with candy or chewing gum
5	Symptom often follows or is associated with a history of medication, recent illness, or dental treatment
6	Regardless of the nature of onset, symptoms persist for many years
7	Fear of cancer/ cancerophobia
8	Sensitive to hot and/or spicy foods
9	Symptoms increased by talking or upon stress or fatigue
10	Little effect with NSAIDs, steroid ointments, gargling, tooth brushing etc.
11	Dysgeusia; loss of taste, taste disturbance, such as a bitter or metallic taste
12	Subjective dry mouth /increased thirst
13	Except burning/numbness, pain often accompanied by discomfort sensation (sore mouth, “rubbed with teeth”, “astringent persimmon juice”, “roughness”, “sticky”, e.g)

**Fig. 3:**
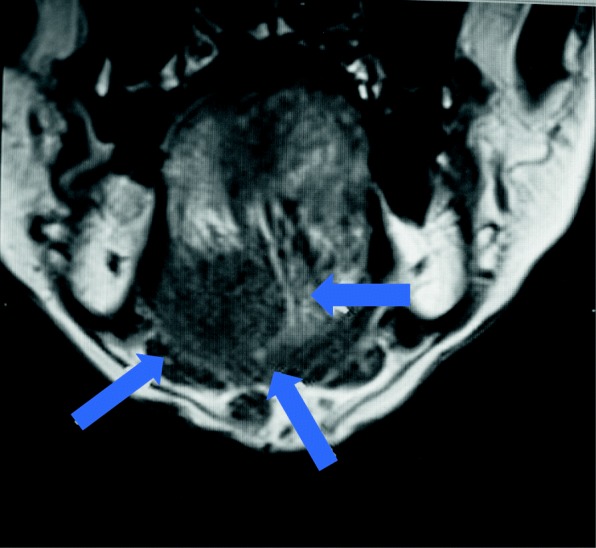
MR imaging a 70 year old male complaining of burning tongue. He was found to have a carcinoma of the left submandibular gland

Our clinical routine usually starts with a review of the medical history, examining extra/intra oral findings, and checking the consistency of subjective symptoms. (Table 1) Then, we perform a general medical examination, do blood tests and salivary measurement, do imaging such as MRI, and CT scan, and give psychological questionnaires [3, 6]. While taking care of elderly patients, who often have multiple systemic diseases and take numerous kinds of medications in addition to experiencing normal physical change from aging, clinicians should be aware of the possibility of underlying malignant tumors (Fig. 3) and dementia [38]. After checking for the above, the final diagnose depends mainly on the patients’ subjective symptoms and history. Most of the complaints of BMS patients’ are focused on their tongue, usually a tingling/burning/numbing sensation or feeling [27]. Symptoms related to the palate, lip, or gingiva are also observed, however, facial skin is not usually affected. Symptoms are often relieved by having a food like chewing gum or candy in the patient’s mouth, and they worsen throughout the day [3].

There are several comorbid oral symptoms other than pain, such as dry mouth and taste disturbance [6]. In addition, BMS has been linked with psychological factors, including stress, depression and anxiety [39]. Cancerophobia, a type of anxiety disorder, was more frequently seen in patients with BMS than in those with other types of orofacial pain [40]. This hints that the “pain” of BMS involves some qualities that evoke life-threatening emotion or restlessness in the patient. Like with other MUOS, BMS patients often do medical institution shopping, but usually are found to have no abnormal findings and thus experience strong frustration. Because of this shopping, delays in the diagnosis of BMS and referral to an appropriate medical institution were frequently reported [41].

## A dilemma in the management of burning mouth syndrome

The management of BMS had been said to be like a ‘jumble of wheat and chaff,’ [42, 43] with little evidence to support or refute the various interventions [44–46]. Moreover, there are “too many reviews and too few trials,” [47] which results in difficulty in choosing the most appropriate approach to therapy for each patient with BMS.

It would be accurate to say that there is no all-powerful treatment that can be effective for all BMS patients, in light of the various underlying conditions. The heterogeneity of this syndrome is the biggest barrier to reaching the best therapy. The nature of BMS is that it is a syndrome that has several causative factors, including some of psychosomatic nature such as chronic pain [24]. Hence, the treatment response of patients differs depending on the predominant individual confounding pathological factors, such as neuropathic components, central sensitization, and psychiatric comorbidities. The problems are intertwined in such a complex way that treatment problems cannot be solved completely by a single therapy.

In addition, no sufficiently effective assessment tool for BMS remission is available. As stated by Albert Einstein, “Not everything that can be counted counts and not everything that counts can be counted” [48]. The suffering of BMS can hardly be explained in Visual Analogue Scale (VAS) scores. Nevertheless, BMS involves not only pure pain sensation but also dysesthesia, such as dryness or dysgeusia. Clinicians, therefore, should carefully consider what a patient claims to be “pain” [29]. To conclude, we need more qualitative assessment methods that provide insight into the patients’ experience of pain instead of depending only on VAS [49].

Like other chronic pain, the treatment outcome of BMS can be explained by placebo effects [50, 51]. In contrast with clinical data such as blood pressure and cell blood counts, VAS does not give precise measurements. With no gold-standard instrument, clinicians struggle to find alternative biomarkers and proper assessment tools for the diagnosis of this long-lasting, complex syndrome.

Another important problem is the assessment of duration and follow-up period [52]. BMS has continuous, long-lasting symptoms that often fluctuate. There is no reliable data on longitudinal outcome or recurrence in existing RCTs for BMS. Considering the nature of BMS, treatment outcome should be assessed after a sufficient period of observation. Retrospective, long-term treatment outcome may be a more critical option. We should observe and analyze the existing data to compare the past and present treatment outcomes in order to improve the patients’ function and quality of life (QoL) [53]. We suggest that real-world data may be more essential than short term RCTs to determine the benefits and limitations of a treatment regimen.

## Currently hopeful treatment strategies

Even though there were many limitations mentioned above, we have high expectations for some treatments for BMS. The efficacy of central neuromodulators (Tricyclic Antidepressants - TCAs, Serotonin and Norepinephrine Reuptake Inhibitors - SNRIs, Selective Serotonin Reuptake Inhibitors - SSRIs, Clonazepam) and cognitive behavioral therapy (CBT) are supported by many studies [3, 9–11, 53, 54] and consistent with our clinical experience.

### Central neuromodulators

In the 1970s, amitriptyline, a TCA, was used for BMS as the first line medication in Japan [53]. Response to TCA needed at least a few days and was not always certain, and side effects emerged quickly and often intensively. If the patients could feel slight improvement, even minor, they were willing to continue the medication and bear its side effects. However, unlike patients with classical trigeminal neuralgia, another type of persistent orofacial pain [13], who usually respond very well to carbamazepine, not all BMS patients can be treated with TCAs. It is important to emphasize here that antidepressants are not always a “magical bullet” for BMS, however, symptom relief could be achieved with careful prescription. Recent research suggests that a change of salivation and QTc interval in cardiology might predict treatment response to amitriptyline [55, 56].

Other than TCAs, SNRIs and SSRIs have shown potential in the treatment of BMS [5, 57–59]. However, they are not always sufficiently effective and some have characteristic side effects and drug interactions (withdrawal symptoms, a little different from TCA) and thus require special caution [60]. In general, the cost-effectiveness of TCA is probably better than SSRIs and SNRIs. Nevertheless, they are useful if their benefits and risks are carefully considered, especially for the elderly [61].

These weak points have inhibited the wide use of neuromodulators. Careful dosing and observation are critical to obtaining the best efficacy with the least side effects. A recent report on functional gastrointestinal disorders and non- gastrointestinal painful disorders recommended using a low to modest dosage of neuromodulators and provide the most convincing evidence of benefit [62], a finding similar to our clinical observation. Additionally, careful attention should be paid to cognitive impairment when long-term medication is provided to elderly patients. To cover these limitations, dopaminergic medications might be helpful in some cases [63, 64]. Nevertheless, they should not be prescribed easily [65].

In addition, Clonazepam - a kind of benzodiazepines (BZs) also used as an antiepileptic, might be a better option than TCAs [66]. It is often used as the first-line medication without severe side effects, except drowsiness, and the patient often feels better shortly. However, its effect usually is temporary, gradually decreases, and contains the risk of dependency, like other BZs. An oral rinse of Clonazepam had high expectations [67], however, in our clinical experience it seems to work successfully only at random. Also, for elderly patients the risk of falling and cognitive disturbances related to systemic BZ prescription must be seriously considered. For the same reason, gabapentinoids (Gabapentin and Pregabalin) should also be prescribed with caution [68].

### Non-pharmacotherapeutic approaches

CBT is one of the effective treatments for burning mouth syndrome. Previous research has shown that the pain severity and discomfort of BMS were improved by CBT targeting cognitive factors [69, 70]. Although the treatment effects were very large and were maintained for 6 months to 12 months, 12 to 16 sessions are necessary to complete a CBT course, which makes it difficult to do for BMS patients due to its high treatment cost.

We recommend here three solutions for reducing the high cost of conducting CBT. The first is using a group format instead of an individual format. Previous research shows that CBT conducted as a form of group treatment and of short duration (1–2 sessions) improved the pain and anxiety of BMS patients [71]. Because no significant difference in effectiveness was shown in comparison with the individual format [72], CBT delivered as group format would be an effective, low-cost alternative solution.

The second solution for BMS patients is limiting the treatment content so that it focuses on specific characteristics. Recently, it was demonstrated that pain-related catastrophizing, a cognitive factor, influences pain severity and oral health-related QoL in BMS [73]. Pain-related catastrophizing maintains and exacerbates chronic pain, thus focusing on pain-related catastrophizing is an important aspect of treatment. Treatment focused on the amelioration of pain-related catastrophizing significantly improved the symptoms of BMS patients [74]. The treatment regimen used in this program consisted of four sessions, showing that CBT can be delivered with low cost by focusing on pain-related catastrophizing.

The third solution is limiting the techniques using in the treatment. Although CBT usually consists of multiple techniques, including psycho-education about disease and treatment and cognitive and behavioral techniques, a treatment regimen containing only psycho-education was shown to be successful for BMS [75]. In this psycho-education program, patients were provided various information about BMS, such as its characteristics, possible mechanisms, and treatment options including medications, which relieved the patients concern about the possible malignant nature of the condition. The importance of maintaining a normal lifestyle despite changes in their symptoms was emphasized. Delivering this extensive information was shown to improve BMS with low cost.

Sleep disorder is another important issue because it is frequently comorbid with BMS, with a prevalence of over 60% [76]. Sleep and chronic pain are bidirectional, thus pain can interfere with sleep and sleep disturbance can exacerbate pain [77]. This high prevalence of sleep disorder might lead to worsening of the symptoms of BMS. CBT-I, an effective treatment for sleep disorder, improved the sleep disturbance and pain symptoms of chronic pain patients [78]. Integrating CBT-I into the usual treatment for BMS would likely enhance the effectiveness of CBT for BMS patients.

Another issue involving BMS is adherence to medication. Although psychotropic medications are effective in the treatment of BMS, a high frequency of non-adherence was found in a population study of psychotropic medications [79]. In BMS, about 15% of the patients stop taking psychotropic medications [80]. These non-adherent patients could be helped by motivational interviewing, which is a CBT technique that can be used to enhance medication adherence [81].

For patients who cannot use any drugs, repetitive transcranial magnetic stimulation (r TMS) may be effective [82, 83]. However, this approach needs an expensive, dedicated machine and requires much more time and effort in the clinic than do the usual pharmacotherapies. Another option is electro-convulsive therapy (ECT), which has shown beneficial results for severe and refractory cases with psychotic features, including a high risk of suicide [84, 85]. Fortunately, few patients are unable to use all of the potential medications, and for the few who can’t we recommend consultation with a psychiatrist.

### Patient education and anxiety management without special therapies

Although CBT is a good option for the management of BMS management, specialist psychotherapists are not always available. Therefore, it is generally difficult to conduct orthodox therapy in the limited- space and time available in a real clinical situation [74]. More importantly, the effect of psychotherapy greatly depends on the capacity of the psychotherapist. Psychotherapy is especially difficult with older patients who have lost their mind plasticity [86].

Clinicians should not feel pressure to diminish all the symptoms of all their patients with BMS or to finish treatment quickly. We not only need to “manage” the symptoms of BMS, but also the improvement of the patients’ QoL. In the general clinical setting, a combination of optimized medication with a short-term supportive psychotherapeutic approach would be a promising solution [2, 53]. Successful disease management cannot be achieved only by pharmacology, but also needs effective communication to build a positive patient-doctor relationship [62].

First, clinicians should be cognizant of patient anxiety, which is not always at a psychopathological level. Patients are often unsettled by chronic oral pain/discomfort of unknown origin [41]. It is important to thoroughly rule out other medical conditions, especially malignancy. These ruling-out processes may relieve the patients’ anxieties and sometimes work as a kind of psychotherapy by themselves [75]. Plain and easy to understand explanation and reassurance that there is no malignancy are necessary. An accurate diagnosis of BMS may also play an important role in relieving a patient’s anxiety/fear of their symptoms. Finally, it can promote the effectiveness of pharmacotherapy of any kind.

Second, it is not always necessary and sometimes impossible to attempt to perfectly diminish pain at once. In a real clinical situation, recovery to normal life should be the priority achievement. “Normal life” does not mean an ideal life without any worries, but can be achieved when a patient can do almost everything necessary to their daily life without being bothered by slight oral symptoms. Even when 99% pain reduction is obtained, some patients continue to be annoyed by the minor residual pain and limit their daily activities. The pain and discomfort of BMS are of such a nature that they evoke emotional distress [21]. Clinicians must understand this characteristic nature of BMS suffering and explain and enhance the role of patients in the medication decision-making process [87] and repeatedly confirm the recovery process. It is also useful for recovery to their normal life to encourage the patient to continue sleep management [77] and adequate physical exercise, such as walking, according to their symptom reduction.

Additionally, the goal of treatment should be shared with patients as well as their family; and a prospective treatment course for BMS should be explained at the first visit to increase motivation for therapy, which can improve the prognosis. However, this is not always successful because of the rather wavelike, up and down, course of some patients’ symptoms. For instance, for some chronic pain patients with co-occurring persistent fatigue, over-predicting the goals in CBT probably leads to poor response [88]. Patients should be informed of the fact that BMS is an intractable pain condition, but that it is not hopeless to obtain complete remission in the future. It is important to choose an appropriate, feasible goal. Supportive understanding from the family is important for BMS patient to prevent discontinuation of treatment, especially so for the elderly. Like other chronic pain conditions, the management of BMS requires empathy, patience, and time from the clinicians, patients, and family.

**Fig. 4:**
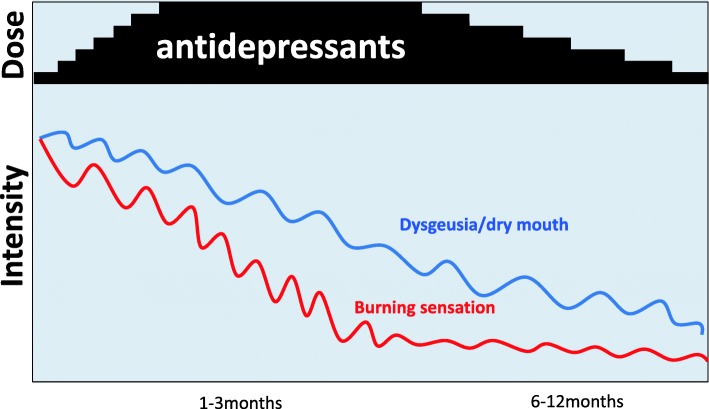
Typical clinical course of a Burning Mouth Syndrome patient treated with Tricyclic Antidepressants

Antidepressants have been shown to sometimes improve the BMS symptoms dramatically, within the first 5 - 7 days, after which the symptoms gradually improve for 1-2 months according to the dose, but perfect remission is not always reached [89]. After some improvement during early treatment, it is very disappointing and irritating for the patients to be confronted with the residual small waves of symptoms, which is no longer called “pain”. Dysgeusia, or subjective dry mouth, has a tendency to improve a little later than pain (burning sensation), so patients tend to complain more about dysesthesia than “pain” in the later period. (Fig. 4) According to our clinical experience, it usually takes minimum of 3–6 months for patients to get to a satisfactory condition and to stabilize. Thorough and careful tapering of the drugs may lead to a good ending of the pharmacotherapy.

Meaningful treatment goals should to be set on level of satisfaction patients will feel as they recover from pain that interferes with their lives and how to adjust to functional disabilities. The focus should not be on the scores of clinical measurements [49]. We should thoroughly understand these real responses to antidepressants for BMS without expecting a “miracle” recovery [89].

It is natural for a patient to feel anxiety about an unstable pain condition of unknown origin [39]. As above mentioned, adding a psychological component to the usual therapeutic regimen based on pharmacotherapy should be considered in the treatment of BMS. These processes are difficult to describe in the context of evidence-based medicine/dentistry.

In summary, BMS remains an enigma and has evolved into a more intractable condition, especially in the elderly. The diagnosis and treatment of BMS remains challenging. There are many problems with the existing treatment data on BMS, and long-term comprehensive assessment and outcome analysis are much needed. It is essential to maintain a supportive attitude to the patients and their family, assuring them of a good prognosis in the near future. Continuing psychological support and the careful use of antidepressants may help with the recovery of the brain function of these patients.

## Abbreviations

BMS: Burning Mouth Syndrome
BZs: Benzodiazepines
CBT: Cognitive behavioral therapy
ECT: Electro-convulsive therapy
IASP: International Association for the Study of Pain
IHS: International headache society
MUOS: medically unexplained oral symptoms
QoL: Quality of life
SNRIs: Serotonin and Norepinephrine Reuptake Inhibitors
SSRIs: Selective Serotonin Reuptake Inhibitors
TCAs: Tricyclic Antidepressants
TMS: Transcranial magnetic stimulation
VAS: Visual Analogue Scale
